# Stability of symptom-based subtypes in Sjogren’s disease

**DOI:** 10.1136/rmdopen-2024-004914

**Published:** 2024-11-24

**Authors:** Joe Scott Berry, Jessica Tarn, John Casement, Dennis Lendrem, Kyle Thompson, Xavier Mariette, Jacques-Eric Gottenberg, Wan-Fai Ng

**Affiliations:** ^1^Translational and Clinical Research Institute, Newcastle University, Newcastle upon Tyne, Tyne and Wear, UK; 2 Clinical and Translational Medicine Research Centre, University of Sunderland, Sunderland, UK; 3 Bioinformatics Support Unit, Newcastle University, Newcastle upon Tyne, UK; 4 Rheumatology, Assistance Publique-Hôpitaux de Paris (AP-HP), Hôpitaux universitaires Paris-Sud – Hôpital Bicêtre, Le Kremlin Bicêtre, France; ^5^Université Paris-Sud, Center for Immunology of Viral Infections and Auto-immune Diseases (IMVA), Institut pour la Santé et la Recherche Médicale (INSERM) UMR 1184, Université Paris-Saclay, Le Kremlin Bicêtre, France; 6 Rheumatology, Hopitaux universitaires de Strasbourg, Strasbourg, France; 7 HRB Clinical Research Facility, University College Cork, Cork, Ireland

**Keywords:** Patient Reported Outcome Measures, Sjogren's Syndrome, Fatigue, Autoantibodies, Classification

## Abstract

**Objectives:**

The Newcastle Sjogren’s Stratification Tool (NSST) stratifies Sjogren’s disease patients into four subtypes. Understanding the stability of the subtypes is vital if symptom-based stratification is to be more broadly adopted. In this study, we stratify patients longitudinally to understand how symptom-based subtypes vary over time and factors influencing subtype change.

**Methods:**

274 patients from the United Kingdom Primary Sjögren’s Syndrome Registry (UKPSSR) with data permitting NSST subtype assignment from two study visits were included. The French Assessment of Systemic Signs and Evolution of Sjogren’s Syndrome (ASSESS) cohort (n=237) acted as an independent comparator. Group analyses of significant differences were performed, with logistic regression models used to assess covariates of subtype stability.

**Results:**

UKPSSR and ASSESS cohorts showed a broadly similar proportion of subjects in each subtype and similar baseline clinical characteristics except body mass index (BMI). Several baseline characteristics differ significantly between the subtypes, most notably anti-Ro status and BMI. Subtype membership was reasonably stable in both cohorts with 60% and 57% retaining subtype. The high-symptom burden subtype was the most stable over time with 70% and 67% retaining subtype. Higher baseline probability score was the greatest predictor of subtype stability with higher C4 levels, antidepressant use, and a higher CCI score also predicting increased stability.

**Conclusion:**

NSST subtype membership remains stable over time in a large proportion of patients. When subtype transition is associated with factors at baseline, it is most strongly associated with an uncertain subtype allocation. Our findings support the hypothesis that symptom-based subtypes reflect genuine pathobiological endotypes and therefore maybe important to consider in trial design and clinical management.

**WHAT IS ALREADY KNOWN ON THIS TOPIC**

- Using patient-reported outcomes (PROs), symptom-based stratification approaches have identified subtypes of Sjogren’s disease.
- Understanding the temporal stability of symptom-based subtypes and PROs is vital, given their potential roles in trial design and therapeutic development.

**WHAT THIS STUDY ADDS**

- This study supports the hypothesis that symptom-based subtypes reflect genuine pathobiological endotypes.
- Our data demonstrate that symptom-based subtype remains stable over time in a large proportion of patients and identifies factors influencing subtype change.

**HOW THIS STUDY MIGHT AFFECT RESEARCH, PRACTICE, OR POLICY**

- Our data aid understanding of the patient experience in Sjogren’s disease and are important to consider if PROs, and PRO-guided stratification, are to be adopted in trial design and clinical management.

## Introduction

 Sjogren’s disease (SjD) is a systemic immune-mediated inflammatory disease. The majority of randomised placebo-controlled trials completed in SjD have not determined the intervention’s clinical efficacy, and no disease-modifying drugs are officially licensed for the indication of SjD.^1^ The clinical manifestations of SjD are diverse and vary greatly between individuals. This heterogeneity is, at least partially, responsible for the variable responses to therapeutics and presents a challenge in trial design and endpoint selection.

In 2019, using data from three European cohorts, Tarn *et al* developed the Newcastle Sjogren’s Stratification Tool (NSST), which stratifies SjD patients into four symptom-based subtypes at the point of care.^2^ The four clinical subtypes identified were classified as low symptom burden (LSB), high symptom burden (HSB), dryness dominant with fatigue (DDF) and pain dominant with fatigue (PDF). The subtypes have distinct molecular profiles and may respond differently to immunomodulatory therapies.^2 3^ Three independent groups have also used this novel symptom-based approach to endotype discovery and identified similar subtypes.^4^,^6^ All four studies identified SjD subgroups with high disease burden, low disease burden and subgroups that were dominated by symptoms of dryness or pain. Furthermore, a longitudinal analysis by Lee *et al* demonstrated these subtypes to be stable over a 5-year follow-up period.^5^ Collectively, these data substantiate the hypothesis that symptom-based subtypes reflect distinct pathobiological endotypes and offer a more precise stratification of SjD patients.

Alongside the development of stratification approaches, there has been increasing interest in the potential utility of patient-reported outcomes (PROs). There is a discordance between objectively measured disease activity and symptoms in SjD, with the pathobiology underpinning many symptoms remaining unclear.^7^ PROs are therefore vital in supporting clinical assessment and laboratory measures to better capture all aspects of SjD and provide a more personalised, meaningful assessment. Indeed, PROs now form an integral part of clinical trials within composite endpoints Composite of Relevant Endpoints for Sjögren’s Syndrome (CRESS) and Sjögren’s Tool for Assessing Response (STAR),^8 9^ in new stratification approaches that integrate additional biological data,^10^ and as part of dual-target, double cohort trials such as the recently published iscalimab and dazodalibep trials.^11 12^ The NSST captures the patient experience by using validated PROs in the form of the EULAR Sjogren’s Syndrome Patient Reported Index (ESSPRI) and Hospital Anxiety and Depression Scale (HADS), which ask patients to assess their symptoms over the last 2 weeks.^13 14^ The allocation of subtype membership is then based on the likelihood (baseline probability score) of the patient belonging to a particular subtype. If the patient’s symptoms are relatively stable then their subtype will remain stable but if a patient’s symptoms change then their subtype will change.

For some patients stratified with NSST, their probability score for different subtypes is similar and therefore their allocated subtype membership is less certain. The subtype membership of such patients may consequently be less stable and prone to alterations with relatively small fluctuations in symptom severity. Better knowledge of how symptom-based subtypes vary longitudinally and the factors that contribute to a change in symptoms, and therefore subtype, are important for understanding the patient experience and are vital if PROs, and stratification based around PROs, are to be used in clinical trial design and therapeutic development. In this study, we stratify the patients in two European cohorts longitudinally using the NSST and examine the temporal stability of the symptom-based subtypes.

## Methods

### Patient cohorts

The United Kingdom Primary Sjögren’s Syndrome Registry (UKPSSR) is a national cohort of clinically well-characterised SjD patients, who fulfil the 2002 American European Consensus Group (AECG) classification criteria.^15^ The UKPSSR holds detailed clinical and laboratory data together with PROs collected prospectively during recruitment using standardised questionnaires. Research ethics approval for the UKPSSR was granted by the North-West Research Ethics Committee, UK. As of 1 January 2023, 274 patients from the UKPSSR had data permitting NSST subtype assignment from two study visits. The follow-up interval was not standardised with a median follow-up interval of 4 years (minimum of 1 year and maximum of 9 years).

The French Assessment of Systemic Signs and Evolution of Sjögren’s Syndrome (ASSESS) cohort was used as an independent comparator cohort. The ASSESS cohort comprises 334 clinically well-characterised SjD patients. Research ethics approval for the ASSESS cohort was granted by the Bichat Teaching Hospital Ethics Committee, France. For the ASSESS cohort, follow-up data were available at 12-month intervals for 5 years. 237 patients had data to assign NSST subtype at baseline and year 5, with 134 patients having complete NSST data at all time points.

For all subjects across both cohorts, the following data were available: age, disease duration, sex, body mass index (BMI), anti-Ro status, lymphocytes, erythrocyte sedimentation rate (ESR), C-reactive protein (CRP), IgG, Schirmer’s tests, EULAR Sjögren’s Syndrome Disease Activity Index (ESSDAI), ESSPRI and HADS.^13 14 16^ In addition, anti-La status, complement component (C3), complement component (C4), EQ-5D UK utility (http://www.euroqol.org), medication history and comorbidity data were also available in the UKPSSR. The cohorts represent a convenience sample and capture the above clinical data when patients attend for clinical review. They are subject to the potential sampling bias associated with this approach including non-representation of the population. However, the involvement of a large number of recruitment sites and the relatively large sampling size should reduce the risk of such bias associated with convenience sampling. The Polypharmacy Score was calculated as the sum of medications at the time of clinic appointment. Details of comorbid conditions were collected prospectively, standardised using International Classfication of Diseases 10 (ICD10) codes, and converted to clinically meaningful categories using Clinical Classifications Software Refined (CCSR) tools. A Chronic Conditions Indicator (CCI) score was calculated using CCSR tools, which were used for downstream analyses. The following medications were assessed for their association with subtype change in the UKPSSR cohort: prednisolone, hydroxychloroquine, azathioprine, mycophenolate, leflunomide, cyclophosphamide, etanercept, infliximab, rituximab, pilocarpine, civemiline, non-steroidal anti-inflammatory drugs, analgesics, antidepressants and hormone replacement therapy.

### Statistical analysis

The NSST uses a logistic regression model to assign patients to symptom-based subtypes based on their responses for ESSPRI-pain, ESSPRI-fatigue, ESSPRI-dryness and HADS. The NSST baseline probability score represents the likelihood of the patient belonging to the symptom-based subtype at initial allocation. Group analyses of significant differences were performed using Wilcoxon analysis and pairwise comparisons were performed using Tukey-Kramer Honestly Significant Differences analysis. Fisher’s exact test was used for categorical data. Logistic regression analysis was performed with the following covariates at baseline to assess their relationship with subtype change: age, sex, BMI, disease duration, anti-Ro status, anti-La status, NSST baseline probability score, ESSDAI score, C3, C4, IgG, WCC, current medications (see list above), CCI and Polypharmacy Score. The relationship between the following covariates and specific subtype changes were assessed in separate logistic regression models: delta anxiety (HAD-A), delta depression (HAD-D), delta fatigue (ESSPRI-fatigue), delta pain (ESSPRI-pain), delta dryness (ESSPRI-dryness), delta EQ-5D UK Utility, delta CCI, delta polypharmacy score. All statistical tests and graphical rendering were performed using the R statistical package and Statistical Analysis System (SAS) JMP Statistical Data Visualisation software 23.

## Results

### Baseline characteristics

Relevant clinical characteristics for both UKPSSR and ASSESS cohorts at baseline are shown in table 1. For both cohorts, the proportion of subjects in each subtype was broadly similar though the ASSESS cohort contained a slightly lower proportion of subjects in the DDF subtype. The BMI was also lower in the ASSESS cohort compared with the UKPSSR cohort across all subtypes. For both cohorts, there was a significant difference in the distribution of seronegative subjects, with a higher proportion of seronegative patients in the PDF and HSB subtypes (p<0.05 for both cohorts).

**Table 1:** Baseline characteristics of the UKPSSR and ASSESS cohorts by baseline subgroup membership. Data displayed are either counts with percentages in brackets or median values with 25th and 75th percentiles in brackets.

		LSB	DDF	PDF	HSB	P value
n	UKPSSR	39 (14.2%)	56 (20.4%)	108 (39.4%)	71 (25.9%)	
	ASSESS	65 (19.5%)	43 (12.9%)	141 (42.2%)	85 (25.4%)	
Age	UKPSSR	59 (49–66)	63 (51–68)	64 (54–70)	57 (50–65)	p=ns (Wilcoxon)
	ASSESS	59 (45–67)	56 (48–64)	58 (51–67)	58 (52–68)	p=ns (Wilcoxon)
Sex	UKPSSR	34F 5M	51F 5M	98F 10M	62F 9M	p=ns (Fisher’s)
	ASSESS	55F 10M	41F 2M	130F 11M	84F 1M	p=0.007 (Fisher’s)
ESSPRI	UKPSSR	2 (1.3–2.7)	5 (4–5.7)	6 (4.7–7.3)	6.7 (5.3–8)	p<0.001 (Wilcoxon)
	ASSESS	2.33 (1.67–2.67)	5 (4.33–6)	6 (5–7.3)	6.7 (5.5–7.7)	p=0.001 (Wilcoxon)
ESSDAI	UKPSSR	2.5 (0–7.25)	3.5 (1–6)	4 (2–8)	5 (2–8)	p=ns (Wilcoxon)
	ASSESS	3 (1–6.8)	3 (2–5)	3 (1–9)	3 (2–7)	p=ns (Wilcoxon)
Anti-Ro positivity	UKPSSR	30 pos9 neg(76.92%)	51 pos5 neg(91.07%)	76 pos32 neg(70.37%)	50 pos21 neg(70.42%)	p=0.01 (Fisher’s)
	ASSESS	44 pos19 neg(69.84%)	31 pos12 neg(72.09%)	68 pos61 neg(52.71%)	46 pos35 neg(56.79%)	p=0.04 (Fisher’s)
Anti-La positivity	UKPSSR	24 pos15 neg(61.53%)	33 pos19 neg2 ND(58.93%)	45 pos50 neg1 ND(41.67%)	38 pos24 neg1 ND(53.52%)	p=0.01 (Fisher’s)
AECG Years	UKPSSR	3 (1–7.5)	4 (1–12.25)	4 (1–9)	3 (1–10)	p=ns (Wilcoxon)
	ASSESS	5 (1–8)	6 (3–10)	5 (2–10)	7 (3–10)	p=ns (Wilcoxon)
BMI	UKPSSR	25.4 (22.5–29)	25.2 (22.3–28.9)	26.3 (23.3–32.3)	27 (23.6–29.7)	p=ns (Wilcoxon)
	ASSESS	22 (20–24)	22 (20–25)	23 (21–28)	25 (21–31)	p=0.0001 (Wilcoxon)
C4	UKPSSR	0.2 (0.16–0.25)	0.21 (0.17–0.26)	0.23 (0.18–0.28)	0.23 (0.17–0.3)	p=ns (Wilcoxon)
EQ-5D UK utility	UKPSSR	0.88 (0.76–1)	0.8 (0.7–0.85)	0.69 (0.59–0.76)	0.62 (0.03–0.69)	p<0.001 (Wilcoxon)
CCI	UKPSSR	3.5 (2.6–5)	4 (2–6)	6 (3–8)	5 (3–8)	p<0.001 (Wilcoxon)
HCQPastPresent	UKPSSR	3 (7.69%)11 (28.20%)	11 (19.64%)15 (26.78%)	23 (21.29%)49 (48.37%)	16 (22.53%)30 (42.25%)	p=0.62 (Fisher’s)
Polypharmacy	UKPSSR	2 (1– 2)	2 (2– 3)	3 (2–4)	3 (2–4)	p<0.001 (Wilcoxon)

AECGAmerican European Consensus GroupASSESSAssessment of Systemic Signs and Evolution of Sjogren’s Syndrome BMI, Body Mass Index; C4, complement factor C4; CCI, Chronic Conditions IndexDDFdryness dominant with fatigueEQ-5D, EuroQual Health Related Quality of Life UK Utility value (five dimensions); ESSDAI, EULAR Sjogren’s Syndrome Disease Activity Index; ESSPRI, EULAR Sjogren’s Syndrome Patient Reported IndexHCQHydroxychloroquineHSBhigh symptom burdenLSBlow symptom burdenPDFpain dominant with fatigue UKPSSRUnited Kingdom Primary Sjögren’s Syndrome Registry

Subtype analysis of baseline demographic characteristics revealed similarities in age, sex, disease duration (AECG years), anti-Ro positivity, ESSPRI and ESSDAI between the cohorts. The significant difference in ESSPRI scores between the subtypes (p<0.001 for both cohorts) reflects the role of the ESSPRI score in contributing to subtype stratification. In both cohorts, the PDF and HSB subtypes trend towards having a higher BMI at baseline and this is significant for the ASSESS cohort (p=0.0001). EQ-5D UK Utility, CCI and Polypharmacy (which were only available for the UKPSSR) were significantly different at baseline for the HSB subtype with CCI and polypharmacy both significantly higher in this subtype (p<0.001) and EQ-5D UK utility significantly lower (p<0.001).

### Overall and subtype stability

**Figure 1:**
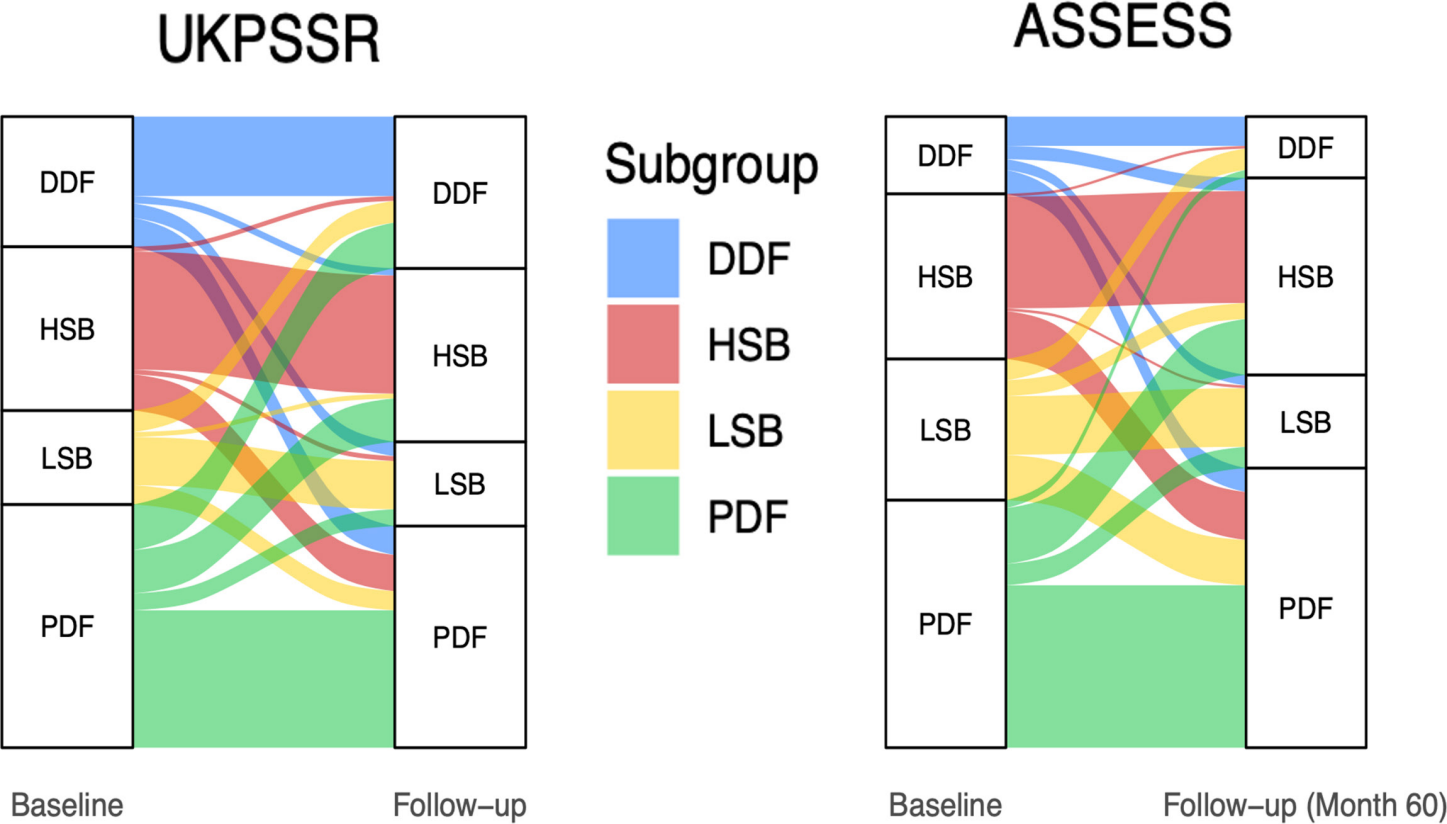
NSST long-term subtype stability. (**A**) UKPSSR cohort: 274 patients classified at baseline and follow-up as an alluvial plot. (**B**) ASSESS cohort: 237 patients classified at baseline and 60 months follow-up as an alluvial plot. Coloured ribbons represent the movement of patients from the initial subgroup to follow-up subgroup. Our analysis highlights that for a large proportion of patients NSST subtype membership remains stable over time. ASSESS, Assessment of Systemic Signs and Evolution of Sjögren’s Syndrome; DDF, dryness dominant with fatigue; HSB, high symptom burden; LSB, low symptom burden; NSST, Newcastle Sjogren’s Stratification Tool; PDF, pain dominant with fatigue; UKPSSR, United Kingdom Primary Sjögren’s Syndrome Registry.

Subtype transition between follow-up visits showed that subtype membership is fairly stable in both cohorts. For the UKPSSR and ASSESS cohorts, subtype membership was maintained in 59.9% and 57.4%, respectively (figure 1 and table 2).

**Table 2:** NSST long-term subtype stability. UKPSSR cohort: 274 patients classified at baseline and follow-up. ASSESS cohort: 237 patients classified at baseline and 60 months follow-up. The table shows the percentage of the entire cohort that retained subtype membership (overall stability) between baseline and final follow-up, along with the percentage of patients that retained subtype membership (subtype stability). The subtype most transitioned to by each subtype and the percentage of the baseline subtype that made this transition are also shown below.

LSB	DDF	PDF	HSB	Overall stability
**UKPSSR**	Subtype membership retained	51.28%	60.71%	55.56%	70.42%	**59.9%**
	Most common transition at follow-up	*DDF*23.07%	*PDF*23.21%	*DDF*20.37%	*PDF*22.53%	
**ASSESS**	Subtype membership retained	41.02%	37.93%	65.59%	67.74%	**57.4%**
	Most common transition at 5- year follow-up	*PDF*32.07%	*PDF*31.03%	*HSB*22.58%	*PDF*29.03%	

ASSESSAssessment of Systemic Signs and Evolution of Sjögren’s Syndrome DDFdryness dominant with fatigueHSBhigh symptom burdenLSBlow symptom burdenPDFpain dominant with fatigueUKPSSRUnited Kingdom Primary Sjögren’s Syndrome Registry

There were also similar patterns of subtype change in both cohorts. In the UKPSSR and ASSESS, the HSB subtype was the most stable with 70.42% and 67.74% maintaining HSB membership, respectively. Of those changing from HSB, the most common transition was to the PDF subtype, with patients continuing to report high pain and fatigue scores. Within the UKPSSR and ASSESS, only 0.73% and 0.42% of the cohorts, respectively, had transitioned from HSB to LSB at the final follow-up. Similarly, only 0.73% in the UKPSSR and 2.53% in ASSESS had made the corresponding transition, from LSB and HSB, at the final follow-up.

In the UKPSSR, the LSB subtype was the least stable with 51.28% maintaining LSB membership at follow-up while 41.02% maintained LSB membership at the 5-year point in the ASSESS cohort. While comparatively lower than HSB and PDF in both cohorts, it still represents a significant proportion of patients maintaining LSB subtype membership at follow-up. The DDF subtype showed the least stability in the ASSESS cohort, though its slightly lower proportion of patients in this cohort should be noted. For those changing from LSB, the most common transitions were to PDF and DDF.

### Predictors of subtype membership stability

**Figure 2:**
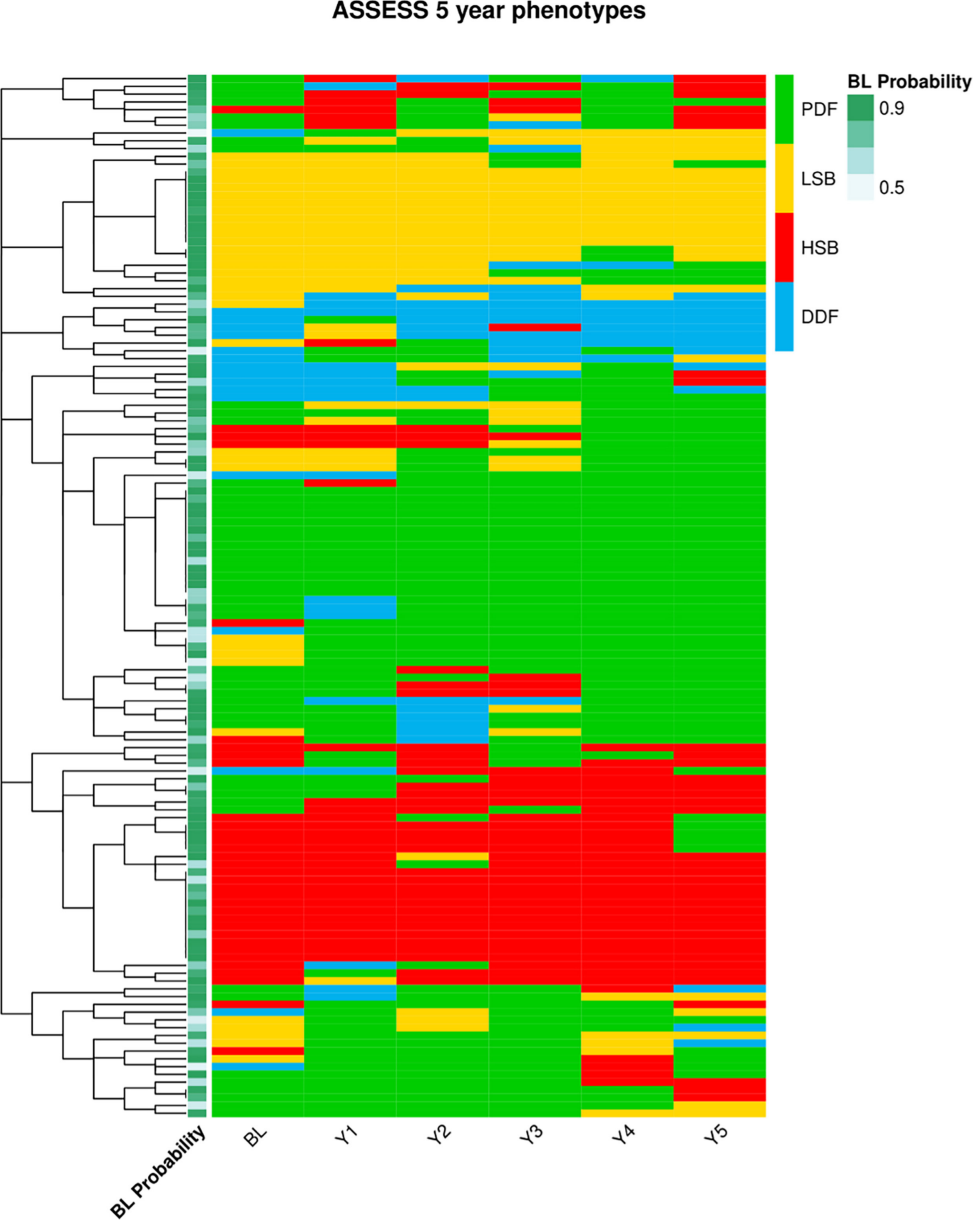
Tracking the ASSESS cohort over 60 months. Figure shows the NSST baseline probability score, the baseline subtype and the subtype at 12-month intervals of patients within the ASSESS cohort. ASSESS, Assessment of Systemic Signs and Evolution of Sjögren’s Syndrome; DDF, dryness dominant with fatigue; HSB, high symptom burden; LSB, low symptom burden; NSST, Newcastle Sjogren’s Stratification Tool; PDF, pain dominant with fatigue;.

We performed regression analysis to define any factors at baseline assessment that are predictors of subtype transition over time. The strongest and most significant predictor of subtype stability is the baseline NSST probability score (table 3). The majority of individuals assigned to a subtype have a probability score of 0.85 at baseline, with 75% in the UKPSSR and 72% in the ASSESS cohort having probability scores of 0.85 (online supplemental figure 1). For some patients, their baseline NSST probability score is lower and therefore their initially allocated subtype membership is less certain. In our analysis, the lower baseline NSST probability predicted less stability and more transition between subtypes. For the ASSESS cohort, the NSST subtype was available at 12-month intervals for 5 years, and the increased transition experienced by patients with lower baseline probability scores over this period can be visualised in figure 2.

**Table 3:** Predictors of subtype change. Table shows factors significantly influencing subtype transition between first and last follow-up.

Candidate predictor	Influence	P value
Baseline NSST probability	Higher baseline probability score is associated with less transition	p=0.0002
Complement component C4	Higher C4 is associated with less transition	p=0.003
Chronic Conditions Index (CCI)	Higher CCI is associated with less transition	p=0.01
Antidepressant usage	Antidepressant usage is associated with less transition	p=0.02
Follow-up interval	Longer follow-up is associated with transition	p=0.03

CCIChronic Conditions IndicatorNSSTNewcastle Sjogren’s Stratification Tool

A higher number of chronic comorbid conditions at baseline (measured by CCI), higher baseline serum levels of complement component C4, antidepressant use and an increased follow-up interval were all also associated with a reduced likelihood of subtype transition (table 3). Complement component C3 was not found to significantly predict subtype change alone (p=0.55) but in conjunction with baseline probability could significantly predict transition (p=0.04) with higher C3 associated with a reduced likelihood of subtype transition. No other factors alone, or in combination with baseline NSST probability, were found to be predictors of subtype change.

## Discussion

Several groups have identified symptom-based subtypes in SjD. In this study, we use two large well-characterised European cohorts to examine the stability of symptom-based subtypes over time and highlight the factors associated with symptom and subtype change. Our analysis of the UKPSSR and ASSESS cohorts showed a broadly similar proportion of subjects in each subtype and, aside from increased BMI across all subtypes in the UKPSSR, no significant differences in baseline clinical characteristics. Although not predictors of change in subtype membership over time, several baseline characteristics differ significantly between the subtypes, most notably anti-Ro status (with increased seropositivity in the DDF and LSB subtypes) and BMI (with increased BMI in the HSB subtype). Proteome analysis suggests NSST subtypes are underpinned by distinct molecular networks, and further analysis of the biological processes underlying differences in seropositivity, BMI and symptom severity between the subtypes is a currently needed task.^17^ Additional data from the UKPSSR also highlighted a higher number of chronic comorbid conditions and increased medication use at baseline in the HSB subtype. This is in keeping with work by McCoy *et al* which highlighted higher prescriptions of NSAID, steroid and immunomodulatory therapies in their HSB group.^6^

With the potential utility of stratification tools increasing, establishing the stability of SjD subtypes assigned to patients is now a vital area of importance. We used NSST to stratify patients longitudinally to assess stability and transition between the subtypes. In a model randomly allocating membership to one of the four subtypes, the chance of remaining in the initial allocated subtype at the final clinic visit is 25%. In the two independent cohorts we assessed, 59.9% and 57.4% did not change subtype between the initial and final clinic visits suggesting that patients report similar levels of pain, fatigue, dryness anxiety and depression during the follow-up period so that the NSST subtypes remain reasonably stable.

With regards to subtype stability, the HSB subtype which experienced the most debilitating symptoms was also the most stable subtype over time. This lack of transition with the significantly higher polypharmacy within this group highlights the unmet clinical need in SjD. It should be noted that a significant proportion of patients (51.28% in the UKPSSR and 41.02% in ASSESS) also maintained LSB subtype membership in both cohorts. Patients within this subtype offer a contrasting perspective of SjD, with LSB while possessing risk factors for extra-glandular manifestations, such as low C4 and lymphopenia.^18^ Both cohorts had few patients who transitioned between LSB and HSB, though they are perhaps the patients most vital to understand further, given these groups comprise the extremes of symptom severity. Our study chronicles patients’ symptoms over several years in two large cohorts, providing a unique view of the patient experience in SjD. Examining symptom-based subtypes may improve our understanding of the mechanisms driving these key symptoms, often the main reasons for seeking medical help from the patients’ perspective. Furthermore, symptom-based stratification may help clinical trials identify patients who would benefit from a particular therapeutic. Indeed, reanalysis of three different phase 3 clinical trials that failed to meet their primary endpoints suggests the HSB subtype may respond to hydroxychloroquine and tocilizumab,^3 17^ while the DDF subtype may respond to rituximab.^2^

Our study is also among the first to demonstrate factors associated with symptom-subtype stability, with the NSST probability score the greatest predictor of subtype stability in this study. It is intuitive that individuals with a lower probability score at baseline, indicating less certain subtype membership, are more likely to change subtypes over time. Individuals with a lower probability score may represent patients who were in transition between subtypes or those who could not be classified accurately using the NSST. Other predictors of subtype stability were higher serum C4 levels, antidepressant use and a higher CCI. C4 has important roles in the clearance of immune complexes, suppressing inflammation and maintaining self-tolerance.^19^ Interestingly, higher levels of serum C4 have also been associated with depression and found to be reduced after antidepressants in some studies.^20^ With the increasing association between inflammation and depression, this intriguing finding warrants further research in the context of SjD.

This study is not without limitations; our list of candidate predictors of transition is not exhaustive and there may be other factors that influence transition not yet assessed and not captured due to the interval between follow-up. Furthermore, as an observational study, we are not able to comment on the causality of the predictors in influencing symptom change only on their association with transition. Further study with a larger sample size for each subtype would allow a more detailed assessment of clinical and laboratory predictors of change for individual subtypes, which could aid the identification of novel therapeutic targets to improve symptoms as well as clinical trial design. Continued establishment of longitudinal datasets with more frequent assessment is important to improve our understanding of symptom stability, responses to interventions and whether the transition from the milder to the more severe NSST subgroups reflects disease progression, disease flare or cycling between disease states. The strategy of recording electronic PROs frequently has been shown to be very beneficial in oncology, with improvements in symptom control, quality of life, time on treatment and survival.^21 22^ Studies of data collection using digital applications in SjD are ongoing and will give further insights into the feasibility and advantages of such strategies.^23 24^

## Conclusion

This study demonstrates that SjD patients report similar levels of pain, fatigue, dryness, anxiety and depression to the extent that the NSST subtype membership remains stable over time in a large proportion of patients. We show that subtype transition is associated with several factors at baseline, most strongly with an uncertain subtype allocation. Our findings support the hypothesis that symptom-based subtypes reflect pathobiological endotypes, and therefore, stratified approaches to drug development and clinical management may be essential.

## supplementary material

10.1136/rmdopen-2024-004914 online supplemental file 1

## Data Availability

All data relevant to the study are included in the article or uploaded as supplementary information.
