# Supplementary material for: Stability of symptom-based subtypes in Sjogren’s disease
Source: RMD Open. 2024 Nov 24;10(4):e004914. doi: 10.1136/rmdopen-2024-004914 (PMC11590857; doi:10.1136/rmdopen-2024-004914)
Supplement: online supplemental file 1 [file rmdopen-10-4-s001.pdf]

Supplementary materials

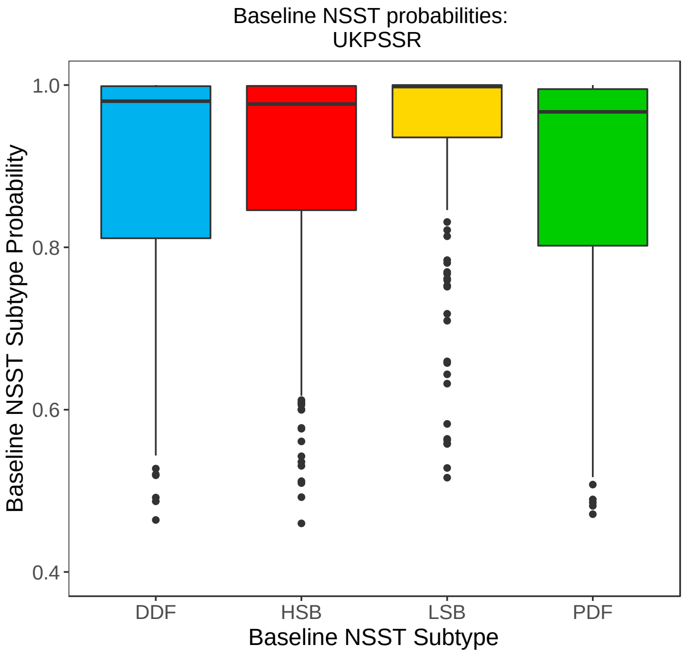

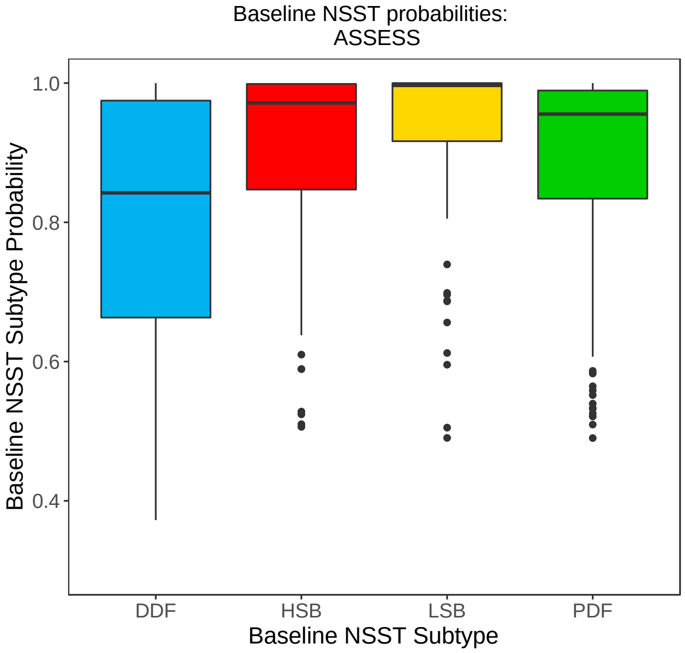

Supplementary figure 1a and 1b. The baseline NSST probability score distribution with the UKPSSR and ASSESS cohorts. Most individuals assigned to a subtype have a probability score of ≥ 0.85 at baseline, with 75% in the UKPSSR and 72% in the ASSESS cohort having probability scores of ≥ 0.85.
